# Black juice in the dark: Pollination of dark‐nectared *Jasminanthes mucronata* (Apocynaceae) by nocturnal hawkmoths

**DOI:** 10.1002/ecy.70370

**Published:** 2026-04-01

**Authors:** Soma Chiyoda, Ko Mochizuki, Atsushi Kawakita

**Affiliations:** ^1^ The Botanical Gardens, Graduate School of Science, The University of Tokyo Tokyo Japan; ^2^ Present address: Misaki Marine Biological Station, Graduate School of Science The University of Tokyo Kanagawa Japan

**Keywords:** Apocynaceae, Asclepiadoideae, colored nectar, East Asia, insect pollination, Japanese white‐eye, pollinaria

While nectar is generally a transparent and colorless liquid, approximately 70 flowering plant species, with at least 15 independent origins, secrete colored nectar (Hansen et al., 2007). Colored nectar is often associated with diurnal pollination by vertebrates such as birds and geckos, and most previous research has focused on such systems (Hansen et al., 2007; Johnson et al., 2006). A few insect‐pollinated plants also secrete colored nectar (Cai et al., 2022; Funamoto, 2023), but these species are also plants with diurnal pollinators. For example, *Stemona tuberosa*, which produces red‐colored nectar, nectar secretion starts in the evening before the flowers open, but pollination is ultimately done by diurnal saprophagous flies (Cai et al., 2022). Consequently, although colored nectar has been discussed as a putative display for diurnal flower visitors, the presence of nocturnal pollinators that visually search for nectar sources, such as moths, raises the possibility that colored nectar may also occur in nocturnal pollination systems.

The subfamily Asclepiadoideae (Gentianales, Apocynaceae) contains several genera with colored nectar, including *Hoya*, *Gymnema*, *Jasminanthes*, *Stephanotis*, and *Vincetoxicum* (Hansen et al., 2007; Liede‐Schumann et al., 2022; K. Mochizuki, personal observation). Of these, the genus *Jasminanthes* Blume includes several species that produce black nectar (Makino, 1904; Rodda, 2019; Tran et al., 2018; Yeoh et al., 2013; Yokogawa, 2022). *Jasminanthes mucronata* is one such species. Distributed in the warm temperate forests of southern China, Taiwan, and Japan (Makino, 1904; Rodda, 2019), it bears white fragrant flowers filled with “black juice” (Makino, 1904; Figure 1a–c). The species' overall floral morphology and fragrance are broadly consistent with moth pollination syndrome (Willmer, 2011). Yamashiro (2003) observed that pollinaria of this species were carried by bumblebees and geometrid and erebid moths, but these observations were based on a short‐term study of a single population, leaving the identity of the primary pollinator unclear. Therefore, we investigated the pollination biology of *J*. *mucronata* to test whether colored nectar occurs in nocturnal pollination systems. We conducted field research in June of 2014–2025 at six sites in Japan (Appendix S1: Section S1, Table S1).

**FIGURE 1:**
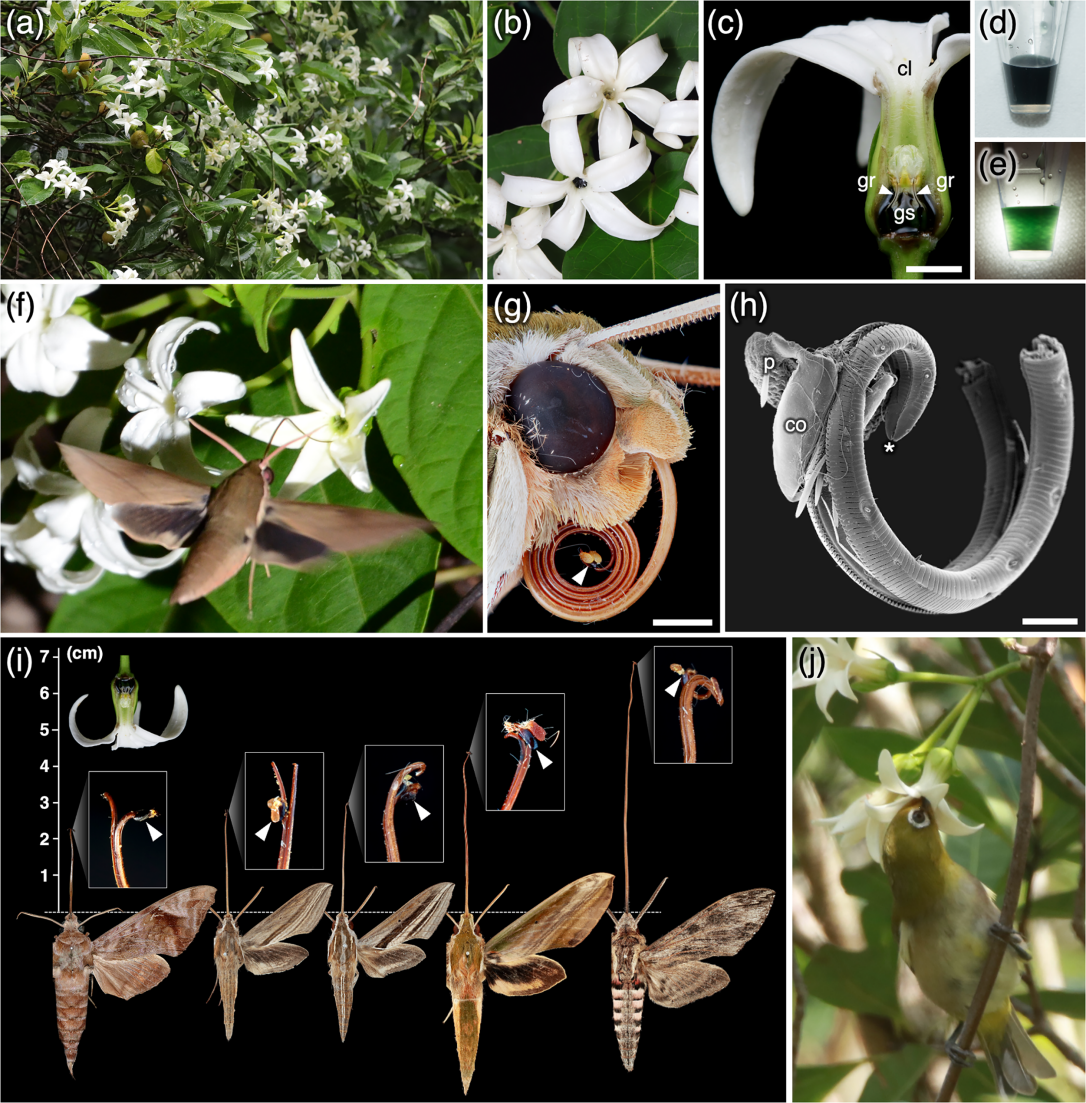
*Jasminanthes mucronata* and its floral visitors. (a) Inflorescences of *J*. *mucronata*. (b) Nectar‐filled flower (bottom) and empty flower (top). (c) Floral structure of *J*. *mucronata*. The front half of the corolla is removed to show the internal morphology. Black nectar is pooled at the base of the corolla tube. (d, e) Nectar of *J*. *mucronata* in a microtube, observed under transmitted light in (e). (f) *Theretra clotho* visiting *J*. *mucronata*. (g) *T*. *nessus* with a single pollinarium (arrow) attached to the tip of its proboscis. (h) SEM image of a pollinarium attached to the proboscis tip of *T*. *japonica*. Asterisk indicates the proboscis tip. (i) Five hawkmoth species with pollinaria attached to the tip of the proboscis. From left to right: *Acosmeryx castanea*, *T*. *japonica*, *T*. *oldenlandiae*, *T*. *nessus*, and *Agrius convolvuli*. White boxes show magnified views of the proboscis tips. Flower of *J*. *mucronata* shown at the upper left at the same scale. Arrows indicate pollinaria. (j) Japanese white‐eye visiting *J*. *mucronata*. cl, corolla; co, corpusculum; gr, guide rail; gs, gynostegium; p, pollinium. Scale bars: (c) = 5 mm; (g) = 2 mm; (h) = 200 μm. Photographs were taken on 18 June 2020 at Minamiise (Site 2) for (a), 19 June 2020 at Kumano (Site 4) for (f), 7 June 2025 at Kochi (Site 6) for (g), and 20 June 2021 at Minamiise (Site 2) for (j). Photographs were taken by Soma Chiyoda (a–c, g–j), Ko Mochizuki (d, e), and Atsushi Kawakita (f).

*J*. *mucronata* flowers began to open gradually after sunset, becoming accessible to nocturnal visitors during the night, although the timing of full opening appeared to vary somewhat among different inflorescences on the same plant (Appendix S1: Figures S1–S3). The nectar appeared black in ambient light but was dark green under strong transmitted light (Figure 1d,e). The volume of nectar per flower was 62.4 ± 30.16 μL (mean ± SD; *n* = 20), the sucrose‐equivalent sugar concentration was 13.7% ± 3.08% Brix (mean ± SD; *n* = 20), and the length of the floral tubes was 13.4 ± 1.6 mm (mean ± SD; *n* = 16; Appendix S1: Section S1, Tables S2 and S3).

We first observed flower visitors at night. We observed 24 moth visits, 16 of which were by nocturnal hawkmoths of at least five species (Figure 1f; Appendix S1: Figure S4a–f, Table S4). Flower visitations by hawkmoths were confirmed at five of the six sites. The hawkmoths appeared to extend their probosces for nectar while hovering. Three hawkmoth individuals and four settling moth individuals visiting flowers could be captured via netting, but they were not carrying pollinaria. It was challenging to capture hawkmoths consistently by netting, and the number of individuals captured was particularly low relative to the frequency of observed flower visits. Therefore, we also conducted light trapping and visual observations on *Lonicera affinis* to efficiently collect hawkmoths present in the area that might have visited *J*. *mucronata* (Appendix S1: Section S1, Figure S5). By these methods, we collected 16 hawkmoth individuals of five species with pollinaria on their proboscises at two sites, and these pollinaria were identified as *J*. *mucronata* based on their morphology, since no plant species with similar pollinaria occurs at the study site (Figure 1g; Appendix S1: Tables S4–S6, Figure S6c−i). One or two pollinaria were attached to the tip of the proboscis, by the pinching force of the corpusculum, which acts as a clamp (Figure 1h; Appendix S1: Table S6, Figure S7). The proboscis lengths of hawkmoths carrying pollinaria varied widely, ranging from 23.0 mm in *Theretra japonica* to 84.7 mm in *Agrius convolvuli* (Figure 1i). Each pollinarium bears two pollinia, but in 10 out of 16 hawkmoth individuals, one or both pollinia were missing. This suggests that the pollinia were successfully deposited on the stigma during visits to multiple flowers, although the possibility that the pollinia were lost due to other causes cannot be entirely ruled out. Both short‐ (<3 cm) and long‐ (>7 cm) tongued hawkmoths had pollinaria with detached pollinia. We experimentally demonstrated that the proboscis of an *Acosmeryx castanea* could extract a pollinarium by sliding it along the guide rail of a *J*. *mucronata* flower, and that a pollinium was subsequently detached and deposited near the stigma (Videos S1 and S2), confirming the potential role of hawkmoths as pollen vectors.

We also conducted observations during the daytime, and observed flower visitations by Japanese white‐eyes, *Zosterops japonicus*, at one site. The white‐eyes repeatedly inserted their beaks into flowers to feed on nectar (Figure 1j; Appendix S1: Figure S4g–j). But, as it was difficult to capture the white‐eyes, we were unable to verify potential pollinarium attachment to the bird's tongue (see Pauw, 1998). We further observed one instance each of a bee, hoverfly, click beetle, and skipper butterfly visiting flowers (Appendix S1: Figure S4k–m). A swallowtail butterfly, *Papilio memnon*, was previously photographed visiting a flower, with its proboscis apparently trapped in the guide rail and unable to be withdrawn; however, no visits by *Papilio* were observed during our observation (Appendix S1: Figure S4n). Of the diurnal visitors, the skipper *Ochlodes ochraceus* had an intact pollinarium attached to the tip of its proboscis (Appendix S1: Figure S6a,b).

Our observations provide direct evidence that hawkmoths and skippers carry pollinaria of *J*. *mucronata*. However, hawkmoths were the most frequent visitors at almost all sites, and 16 individuals from five species had pollinaria attached to their proboscises. The fact that many of these pollinaria had lost one or two pollinia suggests that hawkmoths actually contributed to pollination. From these results, we conclude that hawkmoths are at least one of the principal pollinators of *J*. *mucronata*. Sporadic pollination by hawkmoths has been reported in several asclepiad species (Ollerton et al., 2019), but specialized hawkmoth pollination has so far been documented only in *Schubertia grandiflora* from South America (Amorim et al., 2022). This is the second known case in which hawkmoths are the main pollinators in Asclepiadoideae. In general, hawkmoth‐pollinated flowers are characterized by long, narrow corolla tubes (Johnson et al., 2017), which match the length of the hawkmoths' proboscis. In many hawkmoth‐pollinated plants, the anthers and stigma are located near the entrance of the corolla tube or extend from it, and the pollen is attached to the moth's head or other body parts around the base of the proboscis (Johnson et al., 2017). However, the proboscis lengths of hawkmoths carrying the pollinaria of *J*. *mucronata* ranged from 23.0 to 84.7 mm, which were much longer than the floral tube length of *J*. *mucronata*, 13.4 ± 1.6 mm (Figure 1i). Another species in which hawkmoths serve as the main pollinators, *S*. *grandiflora*, also has a relatively short floral tube (22.6 ± 1.8 mm) and the pollinaria are carried on the proboscises of long‐tongued hawkmoths that exceed 6 cm (Amorim et al., 2022). Although the range of proboscis lengths of pollinator hawkmoths differs between the two species, both asclepiads exhibit successful hawkmoth pollination despite their short floral tubes. Since *J*. *mucronata* (tribe Marsdenieae) and *S*. *grandiflora* (subtribe Gonolobinae) are phylogenetically distinct (Ollerton et al., 2019), these floral traits have likely evolved in parallel. In Asclepiadoideae, the gynostegium, the structure formed by the fusion of the stamens and pistil, is located at the base of the corolla, and the pollinaria and stigma are concealed within it so that pollen transfer is always achieved by the tip of the pollinators' body (Kunze, 1991). Thus, asclepiads may be predisposed to being pollinated by long‐tongued hawkmoths despite having short corolla tubes.

It should not be overlooked that white‐eyes were repeatedly observed visiting flowers at Site 2. Although white‐eyes are known to visit various flowers in East Asia (Funamoto, 2019), this is the first record of them visiting asclepiads. Reports of bird visits to Asclepiadoideae flowers are extremely scarce (Pramsohler & Hilpold, 2012), and pollination by birds has been documented in only a single species, *Microloma sagittatum* from South Africa (Pauw, 1998). In *M*. *sagittatum*, pollinaria are transferred on the sunbirds' tongues. Although it was technically difficult to verify in this study, the possibility that white‐eyes might transfer *J*. *mucronata* pollinaria should be investigated in future studies.

Overall, this study presents a valuable case demonstrating that a pollination system mediated primarily by nocturnal hawkmoths can occur in a plant with colored nectar. Pollination of colored‐nectar flowers by hawkmoths has been reported in *Calliandra calothyrsus* (Fabaceae), but in that species, bats are also thought to contribute to pollination (Hernández, 1991); thus, *J*. *mucronata* represents the first known case in which nocturnal insects appear to be the principal pollinators. Hawkmoths can perceive color in mesopic and scotopic vision environments that are too dark for humans (Sponberg et al., 2015), and forage for nectar using visual guides such as floral color patterns and contrasts (Stöckl & Kelber, 2019). It is possible that the contrast of dark nectar against the white corolla of *J*. *mucronata* can be discerned by hawkmoths even in the dark, potentially acting as an honest signal (Hansen et al., 2007).

On the other hand, the short floral tube of *J*. *mucronata* does not exclude flower visitors other than hawkmoths, such as butterflies and settling moths. Thus, whether the black nectar of *J*. *mucronata* represents a specific adaptation to hawkmoths should be discussed with caution, as it may instead reflect adaptation to both diurnal and nocturnal pollinators. White, short‐tubular flowers are common across the genus *Jasminanthes* (Rodda, 2019) and among related genera such as *Gymnema* and *Stephanotis* (Liede‐Schumann et al., 2022), some species of which possess dark‐colored nectar, while others have colorless nectar. A comparative analysis of pollination systems and nectar color among species of these genera would shed further light on the poorly understood evolution of colored nectar.

## CONFLICT OF INTEREST STATEMENT

The authors declare no conflicts of interest.

## Supporting information


Appendix S1.

Video S1.

Video S2.

Video S1 Metadata.

Video S2 Metadata.
