# Supplementary material for: Black juice in the dark: Pollination of dark‐nectared Jasminanthes mucronata (Apocynaceae) by nocturnal hawkmoths
Source: Ecology. 2026 Apr 1;107(4):e70370. doi: 10.1002/ecy.70370 (PMC13041518; doi:10.1002/ecy.70370)
Supplement: Supplementary file 1 — Appendix S1. [file ECY-107-e70370-s002.pdf]

Appendix S1 for the paper "Black juice in the dark: Pollination of dark-nectared *Jasminanthes mucronata* (Apocynaceae) by nocturnal hawkmoths" authored by Soma Chiyoda, Ko Mochizuki, and Atsushi Kawakita in *Ecology.*

Section S1: *Detailed field observation methods*

Field observations were conducted in natural populations of *Jasminanthes mucronata* at six sites in Japan: 1) Shizuoka-shi, Shizuoka Prefecture (35.0074°N, 138.3396°E, 410 m above sea level [asl]), 2) Minamiise-cho, Mie Prefecture (34.3229°N, 136.6183°E, 40 m asl), 3) Minamiise-cho, Mie Prefecture (34.2678°N, 136.4887°E, 70 m asl), 4) Kumano-shi, Mie Prefecture (33.8408°N, 135.9208°E, 290 m asl), 5) Tomogashima Island, Wakayama Prefecture (34.2813°N, 135.0067°E, 90 m asl), 6) the Kochi Prefectural Makino Botanical Garden, Kochi-shi, Kochi Prefecture (70–120 m asl). At Site 6, we conducted observations that included individuals planted in the botanical garden; however, the garden is located in an area where *J. mucronata* naturally occurs. Both diurnal (25.42 h) and nocturnal (50 h) observations were conducted in June of 2014–2025 (Table S1). The periods from 5:00 to 17:00 and from 17:00 to 5:00 were considered daytime and nighttime, respectively.

Visual observations on *J. mucronata* during the daytime were conducted at a distance of 3–15 m from the inflorescence so as not to disturb flower visitors. Visual observations during the night were conducted approximately 5 m away from the inflorescence, with supplemental red or white light used as needed. Although visual observations revealed that hawkmoths were frequent nighttime visitors, they proved difficult to capture consistently by netting, as the flowers of *J. mucronata* were abundant in the forest canopy and the moths flew rapidly.

For this reason, we conducted light trapping at the same sites to collect flower visitors with positive phototaxis, for a total duration of 17.58 hours. Because light traps can attract moths before they visit flowers (Atwater, 2013), trapping was conducted only after the completion of visual observations to avoid disturbing the flower-visiting behavior. A 74-watt HID light was used for trapping (AKTINA model: Crenata Co. Ltd., Tokyo, Japan). Hawkmoths attracted to the light trap were captured using nets, and their proboscises were examined for the presence of pollinaria. Individuals without attached

pollinaria were released, and we did not quantify the total number of moths attracted to the trap.

In addition, we conducted visual observations of planted *Lonicera affinis* (Caprifoliaceae) at Site 6 to obtain hawkmoths more efficiently in the study area. We observed the flowers for a total of 7.25 hours and collected visiting individuals using nets. *L. affinis* has long corolla tubes and is known to be visited by hawkmoths (Kato, 1987). Compared with *J. mucronata*, the relatively low height of the *L. affinis* shrubs facilitated the capture of visiting insects using nets. The location of the *L. affinis* plantings (33.5488°N, 133.5797°E, 120 m asl) was approximately 30 m from the nearest *J. mucronata* individual. For each captured hawkmoth, we recorded its species, proboscis length, and the presence or absence of *J. mucronata* pollinaria attached to the proboscis.

Nectar volume and concentration were investigated in 2019 at Site 1. Inflorescences containing buds were emasculated using a 1-mm mesh bag at noon on June 15. The inflorescences were checked in the evening (18:00) and newly opened flowers were tagged with masking tape tied to the pedicel. The flowers remained bagged until the following evening, when nectar was sampled using a micropipette with a 200 μL tip. Nectar was preserved in 1.5 mL tubes, and the exact volume was later measured using glass capillaries. The nectar concentration was measured as sucrose equivalent (Brix value) with a handheld refractometer (MASTER-53PM: Atago, Tokyo, Japan) covering a 0.0–53.0% measurement range.

Flower tube length was measured as the distance between the corolla tube opening and the base of the corolla, based on individuals collected at Site 1.

References for the supplementary file

Atwater, M. M. 2013. "Diversity and nectar hosts of flower-settling moths within a Florida sandhill ecosystem." *Journal of Natural History* 47(43–44): 2719–2734. https://doi.org/10.1080/00222933.2013.791944.

Kato, M. 1987. "Coevolution system between angiosperm floras and bumblebees." *Shu-seibutsugaku-kenkyu* 11: 1–13 (In Japanese).

Table S1. Summary of field observations.

Observation site	Date	Duration	Observation methods
Site 5 (Tomogashima)	22 June 2014	1830–2000	Visual observation on *Jasminanthes mucronata*
Site 1 (Shizuoka)	15 June 2018	1200–2000	Visual observation on *Jasminanthes mucronata*
Site 1 (Shizuoka)	16 June 2018	1100–1200	Visual observation on *Jasminanthes mucronata*
Site 1 (Shizuoka)	16 June 2018	1700–2000	Visual observation on *Jasminanthes mucronata*
Site 5 (Tomogashima)	20 June 2018	1600–2100	Visual observation on *Jasminanthes mucronata*
Site 5 (Tomogashima)	21 June 2018	1600–2100	Visual observation on *Jasminanthes mucronata*
Site 5 (Tomogashima)	22 June 2018	1600–2100	Visual observation on *Jasminanthes mucronata*
Site 5 (Tomogashima)	23 June 2018	1600–2100	Visual observation on *Jasminanthes mucronata*
Site 1 (Shizuoka)	15 June 2019	1700–2000	Visual observation on *Jasminanthes mucronata*
Site 4 (Kumano)	19 June 2020	1910–2010	Visual observation on *Jasminanthes mucronata*
Site 1 (Shizuoka)	24 June 2020	1910–2000	Visual observation on *Jasminanthes mucronata*
Site 1 (Shizuoka)	24 June 2020	2000–2200	Light trap
Site 1 (Shizuoka)	25 June 2020	1900–1950	Visual observation on *Jasminanthes mucronata*
Site 4 (Kumano)	28 June 2020	1915–2030	Visual observation on *Jasminanthes mucronata*
Site 4 (Kumano)	28 June 2020	2040–0130	Light trap
Site 4 (Kumano)	29 June 2020	1900–2010	Visual observation on *Jasminanthes mucronata*
Site 2 (Minamiise)	17 June 2021	1540–1600	Visual observation on *Jasminanthes mucronata*
Site 2 (Minamiise)	17 June 2021	1830–2030	Visual observation on *Jasminanthes mucronata*

Site 2 (Minamiise)	17 June 2021	2005–2230	Light trap
Site 2 (Minamiise)	17 June 2021	2300–2320	Visual observation on *Jasminanthes mucronata*
Site 2 (Minamiise)	18 June 2021	1110–1400	Visual observation on *Jasminanthes mucronata*
Site 2 (Minamiise)	18 June 2021	1445–1520	Visual observation on *Jasminanthes mucronata*
Site 3 (Minamiise)	18 June 2021	1930–2130	Visual observation on *Jasminanthes mucronata*
Site 3 (Minamiise)	18 June 2021	2245–2400	Visual observation on *Jasminanthes mucronata*
Site 2 (Minamiise)	19 June 2021	1940–2100	Visual observation on *Jasminanthes mucronata*
Site 2 (Minamiise)	19 June 2021	2100–2240	Light trap
Site 2 (Minamiise)	19 June 2021	2300–2330	Visual observation on *Jasminanthes mucronata*
Site 2 (Minamiise)	20 June 2021	0500–0510	Visual observation on *Jasminanthes mucronata*
Site 2 (Minamiise)	20 June 2021	0640–0750	Visual observation on *Jasminanthes mucronata*
Site 2 (Minamiise)	20 June 2021	1330–1700	Visual observation on *Jasminanthes mucronata*
Site 2 (Minamiise)	20 June 2021	1940–2040	Visual observation on *Jasminanthes mucronata*
Site 2 (Minamiise)	20 June 2021	2100–2210	Visual observation on *Jasminanthes mucronata*
Site 2 (Minamiise)	20 June 2021	2220–2310	Light trap
Site 4 (Kumano)	21 June 2021	1640–1800	Visual observation on *Jasminanthes mucronata*
Site 4 (Kumano)	21 June 2021	1910–2030	Visual observation on *Jasminanthes mucronata*
Site 4 (Kumano)	21 June 2021	2030–2220	Light trap
Site 4 (Kumano)	22 June 2021	1020–1510	Visual observation on *Jasminanthes mucronata*
Site 4 (Kumano)	22 June 2021	1830–2030	Visual observation on *Jasminanthes mucronata*

Site 4 (Kumano)	22 June 2021	2030–2130	Light trap
Site 2 (Minamiise)	23 June 2021	1930–2100	Visual observation on *Jasminanthes mucronata*
Site 2 (Minamiise)	23 June 2021	2100–2130	Light trap
Site 4 (Kumano)	24 June 2021	1930–2200	Light trap
Site 1 (Shizuoka)	26 June 2021	1930–2030	Visual observation on *Jasminanthes mucronata*
Site 6 (Kochi)	5 June 2025	1900–2030	Visual observation on *Jasminanthes mucronata*
Site 6 (Kochi)	5 June 2025	2030–2100	Visual observation on *Lonicera affinis*
Site 6 (Kochi)	6 June 2025	1150–1330	Visual observation on *Jasminanthes mucronata*
Site 6 (Kochi)	6 June 2025	1855–1905	Visual observation on *Jasminanthes mucronata*
Site 6 (Kochi)	6 June 2025	1905–2200	Visual observation on *Lonicera affinis*
Site 6 (Kochi)	7 June 2025	1920–1940	Visual observation on *Jasminanthes mucronata*
Site 6 (Kochi)	7 June 2025	1940–2330	Visual observation on *Lonicera affinis*

Table S2. Nectar volume and sucrose-equivalent sugars concentration of *Jasminanthes mucronata*.

Flower number	Nectar volume (μL)	Sugar concentration (Brix)
#1	61.8	16
#2	88.4	14
#3	93.0	16
#4	92.2	14
#5	100.0	13
#6	88.6	15
#7	77.0	11
#8	43.2	17
#9	79.2	17
#10	72.6	11
#11	43.8	12
#12	31.4	14
#13	13.8	14
#14	35.8	13
#15	15.0	14
#16	33.0	15
#17	24.8	14
#18	55.4	17
#19	97.2	14
#20	101.8	3

Table S3. Floral tube length of *Jasminanthes mucronata*.

Individual number	Tube length (mm)
#1	15
#1	11
#1	11
#1	12
#1	15
#1	14
#1	13
#1	11
#1	13
#1	14
#2	15
#2	15
#2	15
#2	12
#2	13
#2	15

Table S4. Summary of flower visitors observed on *Jasminanthes mucronata* and individuals with pollinaria captured through light trap and visual observations of *Lonicera affinis*.

Observation site	Order	Family	Species	Number of visits to *Jasminanthes mucronata* observed at each site	Number of individuals captured	Number of individuals carrying pollinaria	Observed time (day or night)	Observation methods
Site 1	Lepidoptera	Geometridae	*Thinopteryx crocoptera*	1	1	0	Night	Visual observation on *Jasminanthes mucronata*
Site 1	Lepidoptera	Sphingidae	*Acosmeryx castanea*	-	1	1	Night	Light trap
Site 1	Lepidoptera	Sphingidae	Sphingidae gen. spp.	1	0	0	Night	Visual observation on *Jasminanthes mucronata*
Site 1	Lepidoptera	Erebidae	Erebidae gen. sp.	1	0	0	Night	Visual observation on *Jasminanthes mucronata*
Site 2	Lepidoptera	Sphingidae	*Theretra clotho*	1	1	0	Night	Visual observation on *Jasminanthes mucronata*
Site 2	Lepidoptera	Sphingidae	Sphingidae gen. spp.	6	0	0	Night	Visual observation on *Jasminanthes mucronata*
Site 2	Lepidoptera	Noctuidae	*Thysanoplusia intermixta*	1	1	0	Night	Visual observation on *Jasminanthes mucronata*
Site 2	Diptera	Syrphidae	*Mallota* sp.	1	0	0	Day	Visual observation on *Jasminanthes mucronata*

Site 2	Passeriformes	Zosteropidae	*Zosterops japonicus*	21	0	0	Day	Visual observation on *Jasminanthes mucronata*
Site 3	Lepidoptera	Sphingidae	*Theretra clotho*	1	1	0	Night	Visual observation on *Jasminanthes mucronata*
Site 4	Hymenoptera	unidentified	Apoidea fam. gen. sp.	1	0	0	Day	Visual observation on *Jasminanthes mucronata*
Site 4	Coleoptera	Elateridae	*Melanotus* sp.	1	1	0	Day	Visual observation on *Jasminanthes mucronata*
Site 4	Lepidoptera	Hesperiidae	*Ochlodes ochraceus*	1	1	1	Day	Visual observation on *Jasminanthes mucronata*
Site 4	Lepidoptera	Sphingidae	*Meganoton analis*	2	0	0	Night	Visual observation on *Jasminanthes mucronata*
Site 4	Lepidoptera	Sphingidae	*Theretra clotho*	1	0	0	Night	Visual observation on *Jasminanthes mucronata*
Site 5	Lepidoptera	Sphingidae	*Agrius convolvuli*	1	1	0	Night	Visual observation on *Jasminanthes mucronata*
Site 5	Lepidoptera	Sphingidae	*Marumba gaschkewitschii*	1	0	0	Night	Visual observation on *Jasminanthes mucronata*
Site 5	Lepidoptera	Sphingidae	*Macroglossum saga*	1	0	0	Night	Visual observation on *Jasminanthes mucronata*
Site 5	Lepidoptera	Sphingidae	*Theretra clotho*	1	0	0	Night	Visual observation on *Jasminanthes mucronata*

Site 5	Lepidoptera	Erebidae	*Bastilla arcuata*	1	0	0	Night	Visual observation on *Jasminanthes mucronata*
Site 5	Lepidoptera	Noctuidae	*Thysanoplusia intermixta*	1	0	0	Night	Visual observation on *Jasminanthes mucronata*
Site 5	Lepidoptera	Noctuidae	*Anadevidia peponis*	1	0	0	Night	Visual observation on *Jasminanthes mucronata*
Site 6	Lepidoptera	Sphingidae	*Agrius convolvuli*	-	4	3	Night	Visual observation on *Lonicera affinis*
Site 6	Lepidoptera	Sphingidae	*Psilogramma increta*	-	1	0	Night	Visual observation on *Lonicera affinis*
Site 6	Lepidoptera	Sphingidae	*Ampelophaga rubiginosa*	-	1	0	Night	Visual observation on *Lonicera affinis*
Site 6	Lepidoptera	Sphingidae	*Macroglossum saga*	-	3	0	Night	Visual observation on *Lonicera affinis*
Site 6	Lepidoptera	Sphingidae	*Theretra nessus*	-	25	4	Night	Visual observation on *Lonicera affinis*
Site 6	Lepidoptera	Sphingidae	*Theretra oldenlandiae*	-	3	1	Night	Visual observation on *Lonicera affinis*
Site 6	Lepidoptera	Sphingidae	*Theretra japonica*	-	8	7	Night	Visual observation on *Lonicera affinis*
Site 6	Lepidoptera	Erebidae	Herminiinae gen. sp.	1	1	0	Night	Visual observation on *Jasminanthes mucronata*

Site 6	Lepidoptera	Erebidae	*Mocis* sp.	1	1	0	Night	Visual observation on *Jasminanthes mucronata*

Table S5. Captured hawkmoths and their proboscis length.

Observation site	Sample ID	Species	Proboscis length (mm)	Presence (+) or absence (−) of pollinia attached to the proboscis	Date	Observation methods
Site 1	-	*Acosmeryx castanea*	32.3	+	24 June 2020	Light trap
Site 2	-	*Theretra clotho*	35.2	−	19 June 2021	Visual observation on *Jasminanthes mucronata*
Site 3	-	*Theretra clotho*	37.5	−	18 June 2021	Visual observation on *Jasminanthes mucronata*
Site 5	-	*Agrius convolvuli*	Not measured	−	22 June 2014	Visual observation on *Jasminanthes mucronata*
Site 6	060502	*Agrius convolvuli*	84.7	+	5 June 2025	Visual observation on *Lonicera affinis*
Site 6	060603	*Agrius convolvuli*	73.4	+	6 June 2025	Visual observation on *Lonicera affinis*
Site 6	060702	*Agrius convolvuli*	73.5	+	7 June 2025	Visual observation on *Lonicera affinis*
Site 6	060602	*Agrius convolvuli*	75.7	−	6 June 2025	Visual observation on *Lonicera affinis*
Site 6	060701	*Psilogramma increta*	66.4	−	7 June 2025	Visual observation on *Lonicera affinis*

Site 6	060601	*Ampelophaga rubiginosa*	29.7	−	6 June 2025	Visual observation on *Lonicera affinis*
Site 6	060615	*Macroglossum saga*	32.4	−	6 June 2025	Visual observation on *Lonicera affinis*
Site 6	060703	*Macroglossum saga*	33.8	−	7 June 2025	Visual observation on *Lonicera affinis*
Site 6	060704	*Macroglossum saga*	33.1	−	7 June 2025	Visual observation on *Lonicera affinis*
Site 6	060625	*Theretra nessus*	49.1	+	6 June 2025	Visual observation on *Lonicera affinis*
Site 6	060626	*Theretra nessus*	47.5	+	6 June 2025	Visual observation on *Lonicera affinis*
Site 6	060716	*Theretra nessus*	46.9	+	7 June 2025	Visual observation on *Lonicera affinis*
Site 6	060717	*Theretra nessus*	50.0	+	7 June 2025	Visual observation on *Lonicera affinis*
Site 6	060501	*Theretra nessus*	54.8	−	5 June 2025	Visual observation on *Lonicera affinis*
Site 6	060616	*Theretra nessus*	49.8	−	6 June 2025	Visual observation on *Lonicera affinis*
Site 6	060617	*Theretra nessus*	46.8	−	6 June 2025	Visual observation on *Lonicera affinis*

Site 6	060618	*Theretra nessus*	50.3	–	6 June 2025	Visual observation on *Lonicera affinis*
Site 6	060619	*Theretra nessus*	53.6	–	6 June 2025	Visual observation on *Lonicera affinis*
Site 6	060620	*Theretra nessus*	48.4	–	6 June 2025	Visual observation on *Lonicera affinis*
Site 6	060621	*Theretra nessus*	52.3	–	6 June 2025	Visual observation on *Lonicera affinis*
Site 6	060622	*Theretra nessus*	53.4	–	6 June 2025	Visual observation on *Lonicera affinis*
Site 6	060623	*Theretra nessus*	50.9	–	6 June 2025	Visual observation on *Lonicera affinis*
Site 6	060624	*Theretra nessus*	47.8	–	6 June 2025	Visual observation on *Lonicera affinis*
Site 6	060705	*Theretra nessus*	52.4	–	7 June 2025	Visual observation on *Lonicera affinis*
Site 6	060706	*Theretra nessus*	54.8	–	7 June 2025	Visual observation on *Lonicera affinis*
Site 6	060707	*Theretra nessus*	52.1	–	7 June 2025	Visual observation on *Lonicera affinis*
Site 6	060708	*Theretra nessus*	52.9	–	7 June 2025	Visual observation on *Lonicera affinis*

Site 6	060709	*Theretra nessus*	51.2	−	7 June 2025	Visual observation on *Lonicera affinis*
Site 6	060710	*Theretra nessus*	56.8	−	7 June 2025	Visual observation on *Lonicera affinis*
Site 6	060711	*Theretra nessus*	48.9	−	7 June 2025	Visual observation on *Lonicera affinis*
Site 6	060712	*Theretra nessus*	51.6	−	7 June 2025	Visual observation on *Lonicera affinis*
Site 6	060713	*Theretra nessus*	53.4	−	7 June 2025	Visual observation on *Lonicera affinis*
Site 6	060714	*Theretra nessus*	51.8	−	7 June 2025	Visual observation on *Lonicera affinis*
Site 6	060715	*Theretra nessus*	53.3	−	7 June 2025	Visual observation on *Lonicera affinis*
Site 6	060606	*Theretra oldenlandiae*	30.9	+	6 June 2025	Visual observation on *Lonicera affinis*
Site 6	060604	*Theretra oldenlandiae*	28.2	−	6 June 2025	Visual observation on *Lonicera affinis*
Site 6	060605	*Theretra oldenlandiae*	31.4	−	6 June 2025	Visual observation on *Lonicera affinis*
Site 6	060608	*Theretra japonica*	28.4	+	6 June 2025	Visual observation on *Lonicera affinis*

Site 6	060609	*Theretra japonica*	29.7	+	6 June 2025	Visual observation on *Lonicera affinis*
Site 6	060610	*Theretra japonica*	27.3	+	6 June 2025	Visual observation on *Lonicera affinis*
Site 6	060611	*Theretra japonica*	27.4	+	6 June 2025	Visual observation on *Lonicera affinis*
Site 6	060612	*Theretra japonica*	30.1	+	6 June 2025	Visual observation on *Lonicera affinis*
Site 6	060613	*Theretra japonica*	23.0	+	6 June 2025	Visual observation on *Lonicera affinis*
Site 6	060614	*Theretra japonica*	27.9	+	6 June 2025	Visual observation on *Lonicera affinis*
Site 6	060607	*Theretra japonica*	30.0	−	6 June 2025	Visual observation on *Lonicera affinis*

Table S6. Lepidopteran insects with *Jasminanthes mucronata* pollinaria attached to their proboscises.

Sample ID	Family	Species	Proboscis length (mm)	Number of attached pollinaria (number of remaining pollinia)	Attachment pattern of pollinaria on the proboscis (refer to figure S7)	Observation site	Observed time (day or night)	Observation methods
-	Hesperiidae	*Ochlodes ochraceus*	18.8	1 (2)	Pattern I	Site 4	Day	Visual observation on *Jasminanthes mucronata*
060502	Sphingidae	*Agrius convolvuli*	84.7	1 (1)	Pattern II	Site 6	Night	Visual observation on *Lonicera affinis*
060603	Sphingidae	*Agrius convolvuli*	73.4	1 (1)	Pattern III	Site 6	Night	Visual observation on *Lonicera affinis*
060702	Sphingidae	*Agrius convolvuli*	73.5	1 (0)	Pattern I	Site 6	Night	Visual observation on *Lonicera affinis*
-	Sphingidae	*Acosmeryx castanea*	32.3	1 (1)	Pattern III	Site 1	Night	Light trap
060625	Sphingidae	*Theretra nessus*	49.1	1 (2)	Pattern III	Site 6	Night	Visual observation on *Lonicera affinis*
060626	Sphingidae	*Theretra nessus*	47.5	2 (1, 2)	Pattern IV	Site 6	Night	Visual observation on *Lonicera affinis*
060716	Sphingidae	*Theretra nessus*	46.9	1 (2)	Pattern III	Site 6	Night	Visual observation on *Lonicera affinis*
060717	Sphingidae	*Theretra nessus*	50.0	1 (0)	Pattern III	Site 6	Night	Visual observation on *Lonicera affinis*

060606	Sphingidae	*Theretra oldenlandiae*	30.9	1 (0)	Pattern III	Site 6	Night	Visual observation on *Lonicera affinis*
060608	Sphingidae	*Theretra japonica*	28.4	1 (0)	Pattern III	Site 6	Night	Visual observation on *Lonicera affinis*
060609	Sphingidae	*Theretra japonica*	29.7	1 (2)	Pattern II	Site 6	Night	Visual observation on *Lonicera affinis*
060610	Sphingidae	*Theretra japonica*	27.3	1 (0)	Pattern III	Site 6	Night	Visual observation on *Lonicera affinis*
060611	Sphingidae	*Theretra japonica*	27.4	1 (2)	Pattern II	Site 6	Night	Visual observation on *Lonicera affinis*
060612	Sphingidae	*Theretra japonica*	30.1	1 (2)	Pattern III	Site 6	Night	Visual observation on *Lonicera affinis*
060613	Sphingidae	*Theretra japonica*	23.0	2 (0, 2)	Pattern IV	Site 6	Night	Visual observation on *Lonicera affinis*
060614	Sphingidae	*Theretra japonica*	27.9	1 (2)	Pattern III	Site 6	Night	Visual observation on *Lonicera affinis*

Figure S1

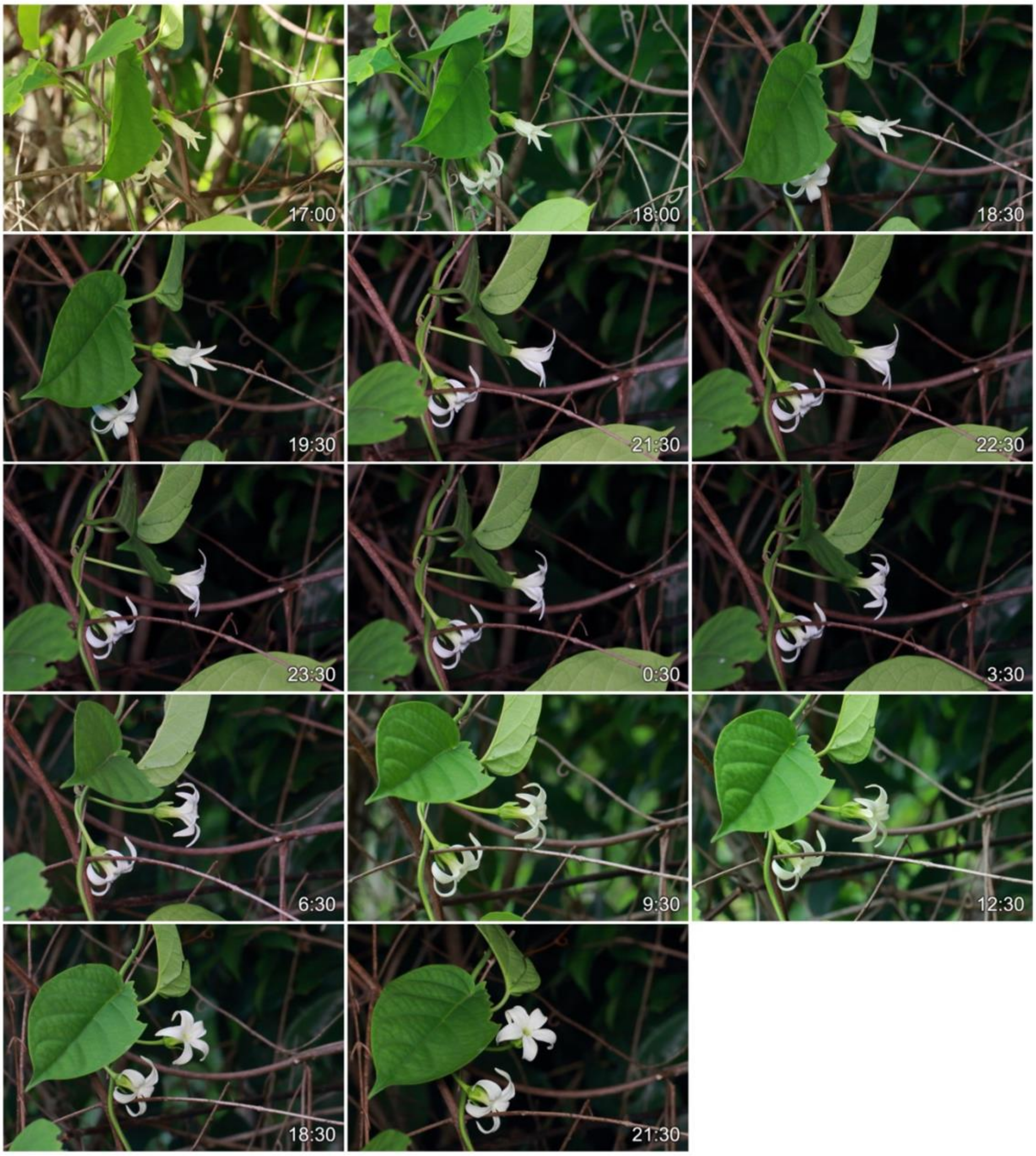

Figure S1. Observation of the inflorescence of *Jasminanthes mucronata*. Photographs were taken during 21–22 June 2021 at Kumano (Site 4) by Soma Chiyoda.

Figure S2

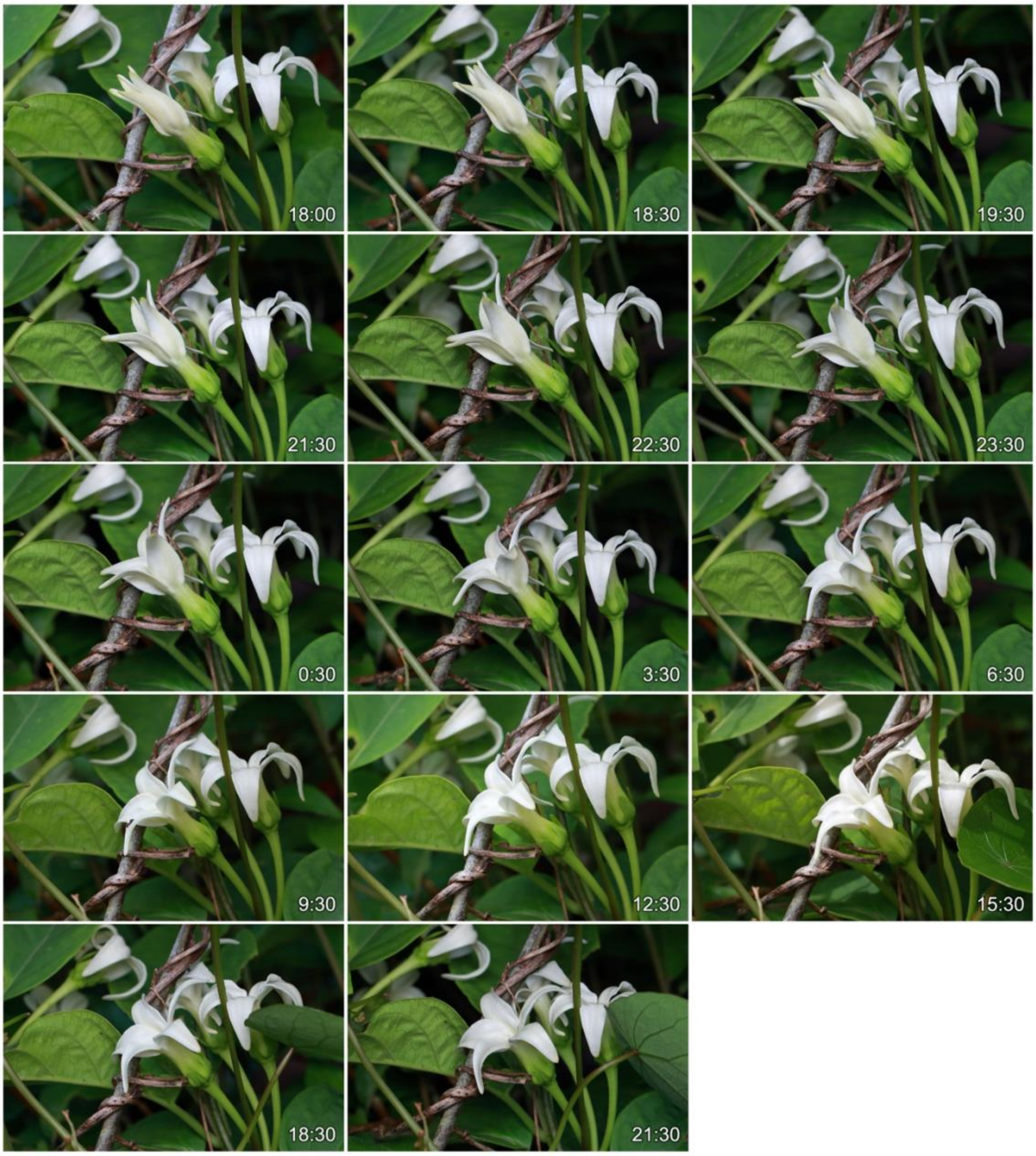

Figure S2. Observation of the inflorescence of *Jasminanthes mucronata*. Photographs were taken during 21–22 June 2021 at Kumano (Site 4) by Soma Chiyoda.

Figure S3

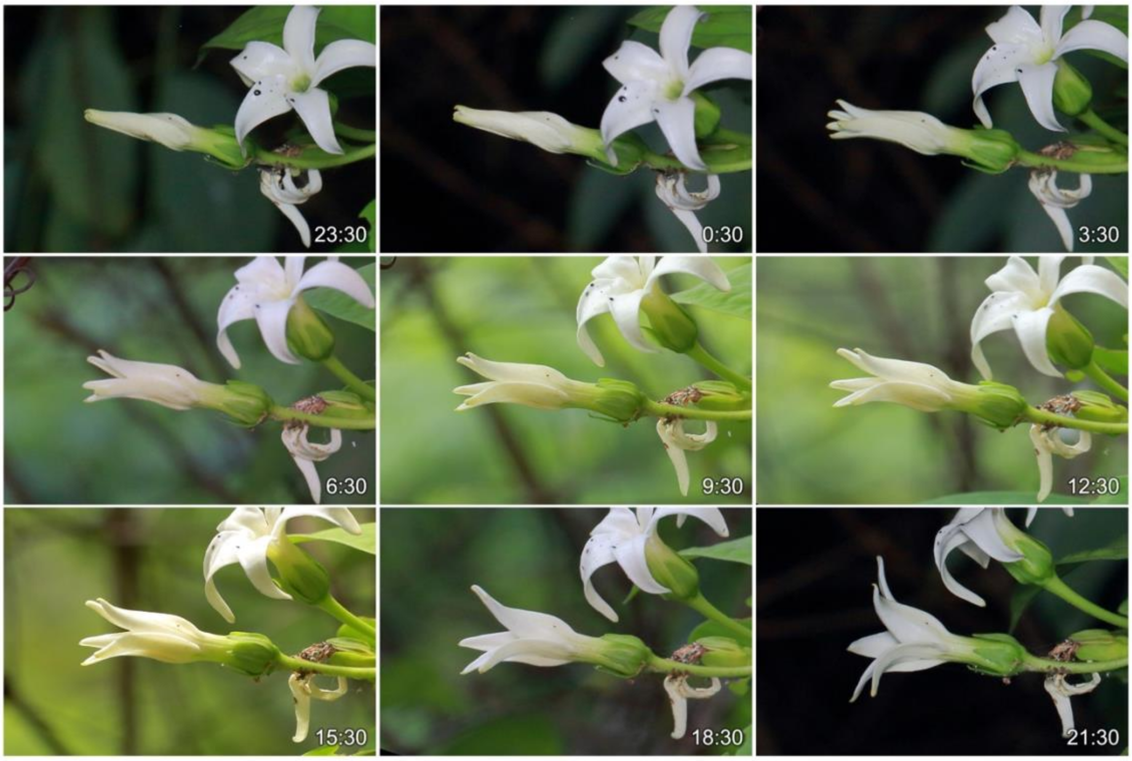

Figure S3. Observation of the inflorescence of *Jasminanthes mucronata*. Photographs were taken during 21–22 June 2021 at Kumano (Site 4) by Soma Chiyoda.

Figure S4

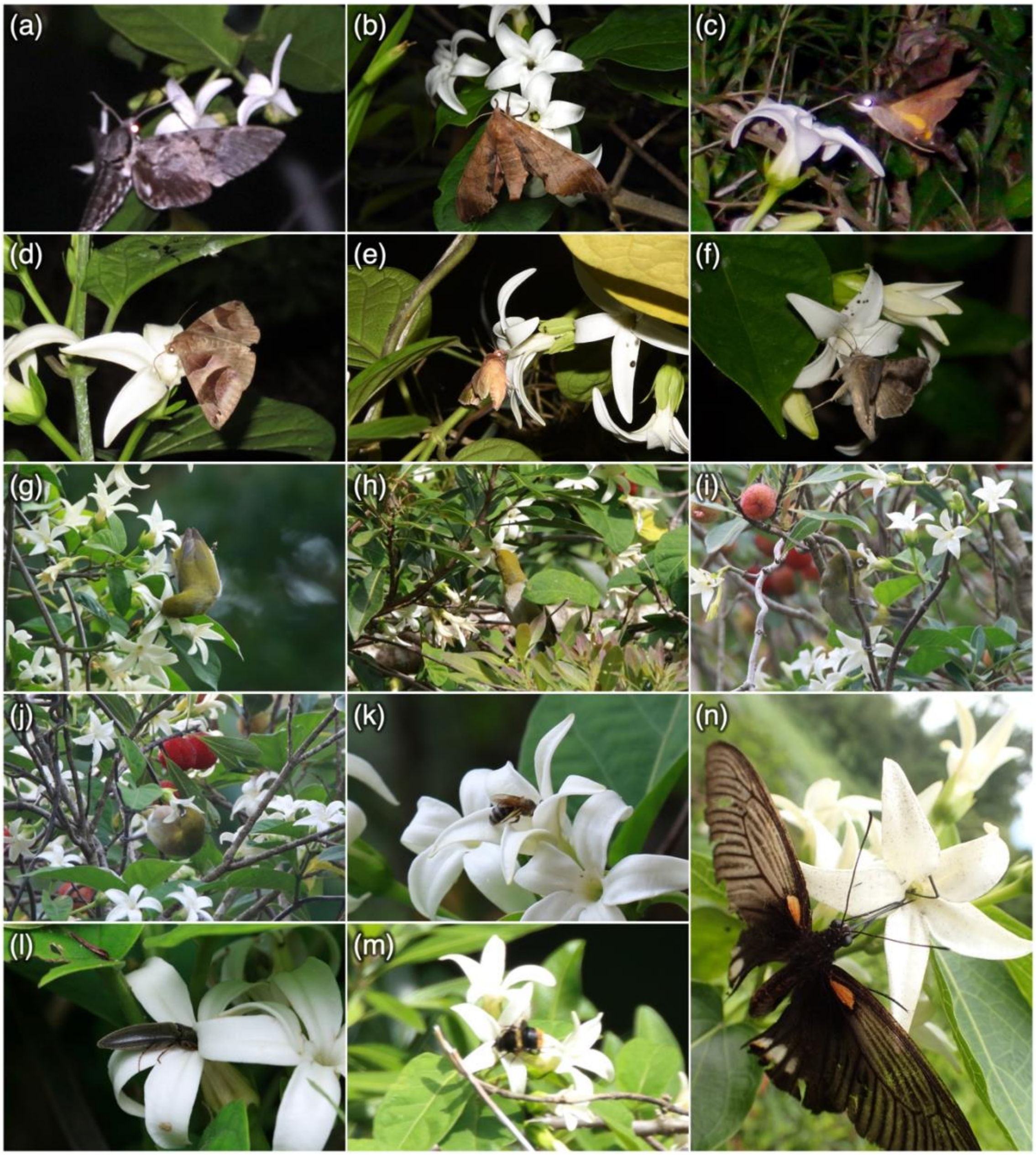

Figure S4. Flower visitors of *Jasminanthes mucronata*. (a) *Meganoton analis*. (b) *Marumba gaschkewitschii*. (c) *Macroglossum saga*. (d) *Bastilla arcuata*. (e) *Thysanoplusia intermixta*. (f) *Anadevidia peponis*. (g–j) *Zosterops japonicus*. (k) Apoidea fam. gen. sp. (l) *Melanotus* sp. (m) *Mallota* sp. (n) A individual of *Papilio memnon* whose proboscis became entangled in a flower. Photographs were taken at night on 29 June 2020 at Kumano (Site 4) for (a), 21 June 2018 at Tomogashima (Site 5) for (b) and (f), 22 June 2014 at Tomogashima (Site 5) for (c, d), and 15 June 2018 at Shizuoka (Site 1) for (e);

during the daytime on 18–20 June 2021 at Minamiise (Site 2) for (g–j), 21 June 2021 at Kumano (Site 4) for (k, l), 20 June 2021 at Minamiise (Site 2) for (m), and unknown date at Kochi (Site 6) for (n). Photographs were taken by Soma Chiyoda (a, g–m) and Ko Mochizuki (b–f). Photograph (n) was provided by the Kochi Prefectural Makino Botanical Garden.

Figure S5

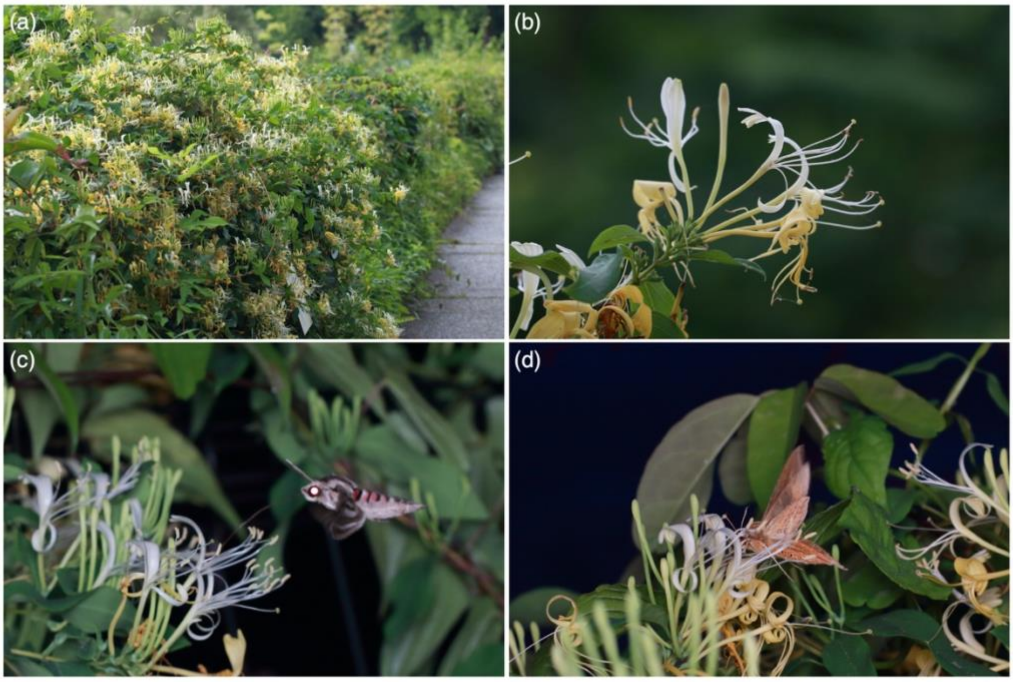

Figure S5. Visual observations on *Lonicera affinis* at Kochi (Site 6). (a) Inflorescences of *L*. *affinis*. (b) Several flowers of *L*. *affinis*. (c) *Agrius convolvuli* visiting *L*. *affinis*. (d) *Theretra japonica* visiting *L*. *affinis*. Photographs were taken on 8 June 2025 for (a, b), 7 June 2025 for (c), and 6 June 2025 for (d). All photographs were taken by Soma Chiyoda.

Figure S6

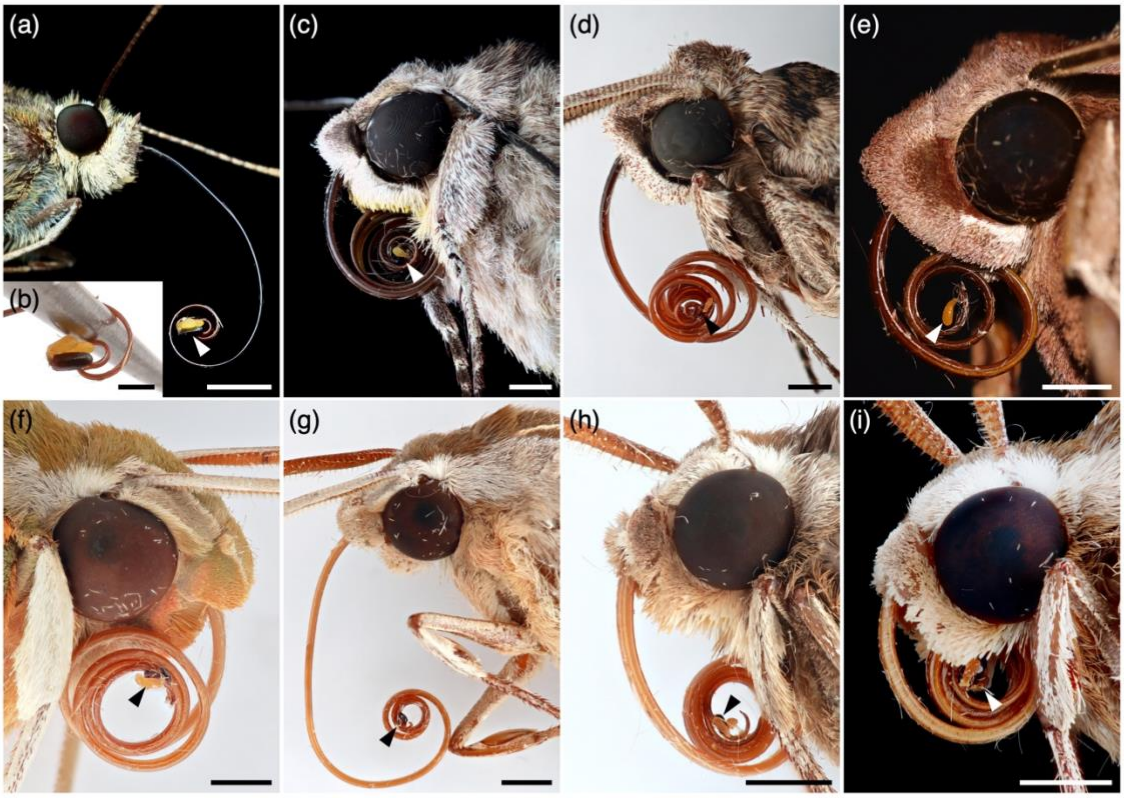

Figure S6. A skipper butterfly and hawkmoths carrying pollinaria of *J. mucronata* on the tip of proboscises. (a) *Ochlodes ochraceus*. (b) Magnified view of the pollinarium. (c, d) *Agrius convolvuli*. (e) *Acosmeryx castanea*. (f) *Theretra nessus*. (g) *T. oldenlandiae*. (h, i) *T. japonica*. Arrows indicate pollinaria. Scale bars: (a, c–i) = 2 mm; (b) = 500 µm. Specimens were collected on 22 June 2021 at Kumano (Site 4) for (a, b), 5 June 2025 at Kochi (Site 6) for (c), 6 June 2025 at Kochi (Site 6) for (d, f–i), and 24 June 2020 at Shizuoka (Site 1) for (e). All photographs were taken by Soma Chiyoda.

Figure S7

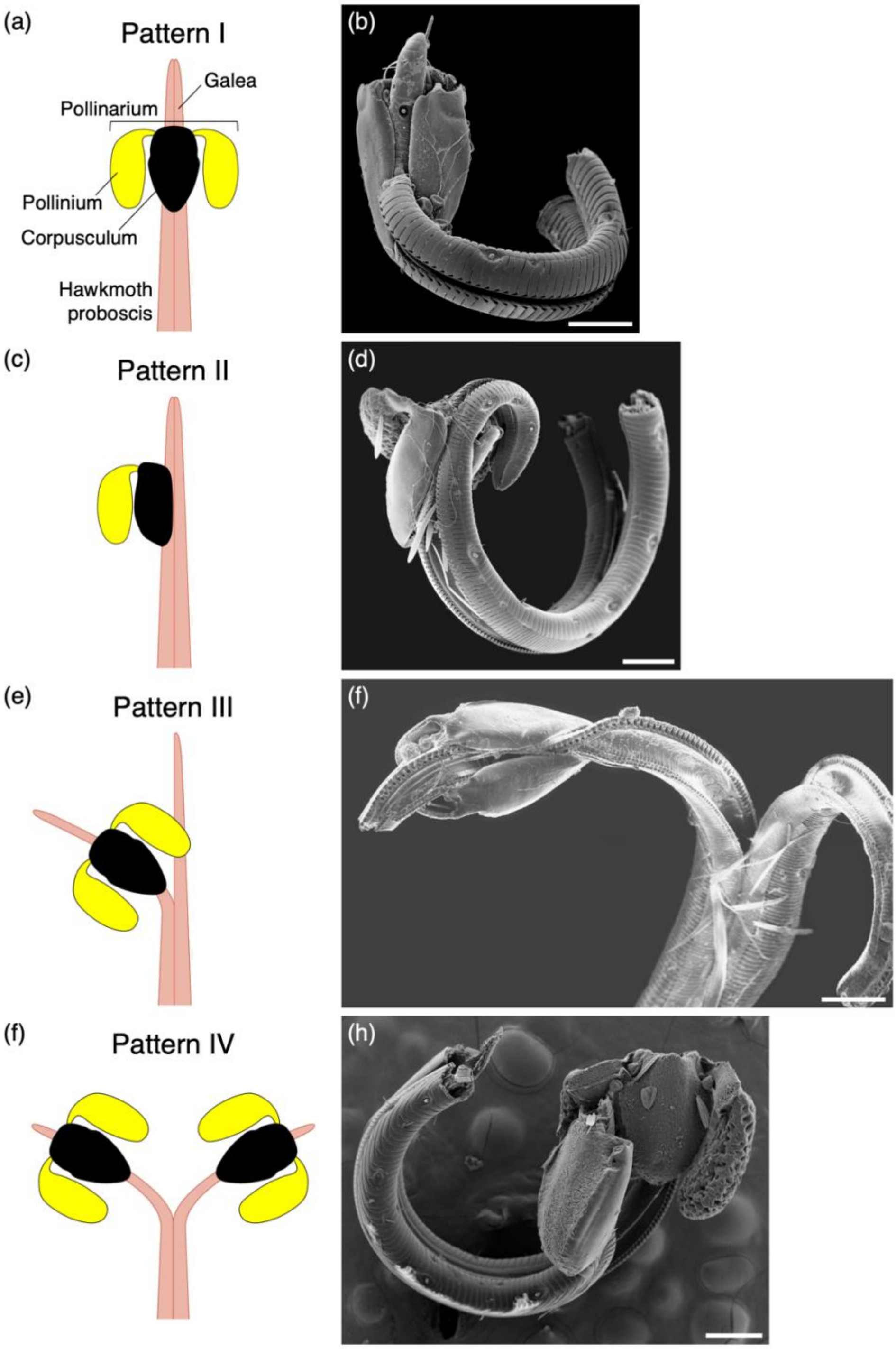

Figure S7. Four attachment patterns of pollinaria on the proboscis of hawkmoths. (a, b) Pattern I: a pollinarium attached at the tip of the proboscis, binding together the paired galeae. (c, d) Pattern II: a pollinarium was attached laterally at the tip of the proboscis, clasping one of the paired galeae. (e, f) Pattern III: a pollinarium was attached to one of

the paired galeae that had split apart at the tip of the proboscis. (g, h) Pattern IV: pollinaria were attached to both of the paired galeae that had split apart at the tip of the proboscis. (a, c, e, g) Schematic diagram. (b, d, f, h) SEM images of the pollinaria attached to the tip of the proboscis. (b) *Agrius convolvuli*. (d) *Theretra japonica*, same as figure 1h. (f) *Acosmeryx castanea*. (h) *T. japonica*. Scale bars: (b, d, f, h) = 200 μm. Specimens were collected on 7 June 2025 at Kochi (Site 6) for (b), 6 June 2025 at Kochi (Site 6) for (d, h), and 24 June 2020 at Shizuoka (Site 1) for (f). All schematic diagrams were created by Soma Chiyoda, and all SEM images were taken by Soma Chiyoda.
