# Supplementary material for: Black juice in the dark: Pollination of dark‐nectared Jasminanthes mucronata (Apocynaceae) by nocturnal hawkmoths
Source: Ecology. 2026 Apr 1;107(4):e70370. doi: 10.1002/ecy.70370 (PMC13041518; doi:10.1002/ecy.70370)
Supplement: Supplementary file 4 — Video S1 Metadata. [file ECY-107-e70370-s003.pdf]

Supporting Information

Black juice in the dark: Pollination of dark-nectared *Jasminanthes mucronata* (Apocynaceae) by nocturnal hawkmoths

Soma Chiyoda, Ko Mochizuki, Atsushi Kawakita

Video S1: An experiment to artificially pull out a pollinarium of *Jasminanthes mucronata* by a proboscis of a hawkmoth, *Acosmeryx castanea*. Note that the individual used in the experiment had the paired galeae split at the tip of the proboscis.
Video credit: Soma Chiyoda
