# Supplementary material for: Black juice in the dark: Pollination of dark‐nectared Jasminanthes mucronata (Apocynaceae) by nocturnal hawkmoths
Source: Ecology. 2026 Apr 1;107(4):e70370. doi: 10.1002/ecy.70370 (PMC13041518; doi:10.1002/ecy.70370)
Supplement: Supplementary file 5 — Video S2 Metadata. [file ECY-107-e70370-s004.pdf]

Supporting Information

Black juice in the dark: Pollination of dark-nectared *Jasminanthes mucronata* (Apocynaceae) by nocturnal hawkmoths

Soma Chiyoda, Ko Mochizuki, Atsushi Kawakita

Video S2: An experiment in artificial pollination by passing a pollinium of *Jasminanthes mucronata* attached to the tip of a proboscis of a hawkmoth, *Acosmeryx castanea*, through the guide rail. The individual used in this experiment was the same as that shown in Video S1.

Video credit: Soma Chiyoda
